# First Detection and Genetic Characterization of Influenza D Virus in Cattle in Spain

**DOI:** 10.3390/vetsci13020130

**Published:** 2026-01-29

**Authors:** Alfredo A. Benito, Luis V. Monteagudo, Sofía Lázaro-Gaspar, Laura Garza-Moreno, Nuria Antón-Baltanás, Joaquín Quílez

**Affiliations:** 1 Exopol S.L., Pol Río Gállego D/14, San Mateo de Gállego, 50840 Zaragoza, Spain; abenito@exopol.com (A.A.B.); slazaro@exopol.com (S.L.-G.); nanton@exopol.com (N.A.-B.); 2 Department of Anatomy, Embryology and Genetics, Faculty of Veterinary Sciences, University of Zaragoza, 50013 Zaragoza, Spain; 3 Research Group A16_23R (Zoonosis and Emerging Diseases of Public Interest), Faculty of Veterinary Sciences, University of Zaragoza, 50013 Zaragoza, Spain; jquilez@unizar.es; 4 Research Agrifood Institute of Aragon (IA2), Faculty of Veterinary Sciences, University of Zaragoza, 50013 Zaragoza, Spain; lgarza@unizar.es; 5 Department of Animal Pathology, Faculty of Veterinary Sciences, University of Zaragoza, 50013 Zaragoza, Spain

**Keywords:** influenza D virus, respiratory pathogens, cattle, occurrence, genetic lineages, Spain

## Abstract

Influenza D virus (IDV) and co-circulating respiratory pathogens were investigated using RT-qPCR in 316 samples collected from 210 Spanish cattle farms with bovine respiratory disease (BRD) outbreaks between July 2023 and September 2024. Thirty-eight IDV-positive samples, from 30 farms across 15 provinces throughout Spain, were identified. IDV was significantly more frequent in bronchoalveolar lavage samples (22.1%) and nasal swabs (13.5%) compared to lung tissues (5%) and other sample mixtures (5%). All IDV-positive specimens exhibited co-infections, with most (94.7%) harboring three to seven viral and/or bacterial pathogens, highlighting the complexity of BRD outbreaks. Cramer’s V analysis revealed moderate IDV association with *Mycoplasma bovis* (V = 0.255) and *Pasteurella multocida* (V = 0.223), and a weaker IDV-bovine coronavirus association (V = 0.202), while IDV association with *Histophilus somni* (V = 0.025) and bovine herpesvirus 1 (V = 0.000) was negligible. Partial sequences of the hemagglutinin-esterase (*HEF*) gene from a subset of 13 IDV-positive samples (Ct < 32) were obtained. This confirmed the presence of the two major genetic lineages detected among cattle in Europe, D/OK and D/660, with D/660 exhibiting higher genetic diversity, as determined by DNAsp 6.12 software. This is the first report of IDV infection in Spanish cattle, confirming the circulation of the D/OK and D/660 lineages within the cattle population.

## Simple Summary

We analyzed 316 samples from 210 Spanish cattle farms suffering respiratory diseases between July 2023 and September 2024 in order to detect Influenza D virus (IDV) and other respiratory pathogens. For IDV, 38 samples (12%) from 30 farms (11.1%) were positive across 15 provinces throughout Spain. IDV was more frequently detected in bronchoalveolar lavage samples (22.1%) and nasal swabs (13.5%) compared to lung tissues (5%) and other sample mixtures (5%). All IDV-positive specimens exhibited other infectious agents too; furthermore, 94.7% harbored three to seven pathogens, demonstrating the complex nature of bovine respiratory diseases. Statistical analysis revealed moderate IDV-*Mycoplasma bovis* and IDV-*Pasteurella multocida* associations, weak IDV-bovine coronavirus and IDV-*Histophilus somni* association and negligible association with bovine herpesvirus 1. In a subset of 13 samples showing a high concentration of IDV, we obtained partial sequences of the hemagglutinin-esterase (*HEF*) gene: sequencing confirmed the presence of the two major genetic IDV lineages previously detected among cattle in Europe, D/OK and D/660. The samples belonging to the D/660 lineage exhibited higher genetic diversity. This is the first report of IDV infection in Spanish cattle, confirming the circulation of both the D/OK and D/660 lineages in the cattle population.

## 1. Introduction

Influenza D virus (IDV) is an emerging pathogen primarily affecting the bovine respiratory system, and it contributes to the multifactorial origin of Bovine Respiratory Disease (BRD), one of the main diseases in the cattle industry, impacting both dairy and calf rearing units all over the world. Both viral pathogens—such as bovine herpesvirus 1 (BHV-1), bovine viral diarrhea virus (BVDV), parainfluenza-3 virus (BPI3V), bovine respiratory syncytial virus (BRSV) and bovine coronavirus (BCV) and bacterial pathogens, including *Mannheimia haemolytica*, *Mycoplasma bovis*, *Pasteurella multocida* and *Histophilus somni*, are known to cause BRD. These agents often interact synergistically in co-infections, thereby exacerbating disease severity in outbreaks [1,2,3]. Environmental and management factors, as well as the immune status of the host, also contribute to the multi-etiological nature of BRD.

BRD generates substantial economic losses in young cattle that have increased in recent decades [4,5]. In the United Kingdom, nearly 70% of cattle farmers experience BRD in their herds, with almost 50% reporting animal losses attributed to the disease [6]. In the United States, BRD is estimated to cost the cattle industry $800 to $900 million annually, primarily due to calf deaths, veterinary expenses, and labor costs [7].

IDV is a novel RNA virus belonging to the family *Orthomyxoviridae*. It was first identified in swine suffering respiratory illness in Oklahoma, USA, in 2011, and it has been detected globally since then [8,9]. Recent studies indicate that IDV is particularly prevalent in cattle populations exposed to high-density management practices such as feedlots. The virus is transmitted through respiratory secretions, and its control is currently a challenge to veterinary practitioners worldwide. The clinical presentation of IDV infections in cattle can range from mild to severe, and diagnosis is typically confirmed through molecular diagnostics such as PCR, which detects viral genetic material in nasal swabs or lung tissue samples [10,11].

Currently, IDV strains can be classified into at least five phylogenetic lineages based on the divergence of the hemagglutinin-esterase fusion (*HEF*) gene, which is the primary target for neutralizing antibodies generated during IDV infection. These lineages include D/OK (D/swine/Oklahoma/1334/2011), D/660 (D/bovine/Oklahoma/660/2013), D/Yama2016 (D/bovine/Yamagata/10710/2016), D/Yama2019 (D/bovine/Yamagata/1/2019) and D/CA2019 (D/bovine/California/0363/2019) [12]. The D/OK and D/660 lineages are predominant in Europe and North America, and multiple re-assortment events between these two lineages have also been detected [8,13,14]. The D/CA2019 lineage has been identified exclusively in the United States, whereas D/Yama2016 and D/Yama2019 are mainly restricted to Asian countries such as China, Japan, and Korea [9,15,16]. In addition, divergent lineages have been proposed in Brazil and in Turkey (the latter tentatively called D/Bursa2013 and characterized by two nucleotide substitutions in *HEF*) [17,18].

Several studies have investigated IDV infections in cattle in European countries since the first description in France in 2012 [19]. Sero-prevalence rates reported range from 80.2% in Luxembourg, 64.9% in Ireland, 47.2% in France and >80% in Italy, suggesting this virus is circulating widely in these countries [20,21,22,23]. In the United Kingdom, IDV has been detected in cattle using molecular techniques, often as the sole viral agent, and consistently in conjunction with one or more bacterial co-infections [24]. IDV infection has been detected by RT-PCR in cattle in Ireland, Italy, Switzerland, Denmark and Sweden [25,26,27,28,29]. To our knowledge, IDV has not been reported in Spain to date. In the current study, samples from cattle showing respiratory symptoms in Spanish farms were submitted to molecular testing in order to investigate the occurrence of IDV in BRD etiology.

## 2. Materials and Methods

### 2.1. Ethical Concerns

Ethics committee approval is not required for this study because it involved non-experimental, routine clinical veterinary practices. These procedures were conducted at the request of animal owners for diagnostic purposes.

### 2.2. Samples

A total of 316 samples from cattle were used. These specimens were submitted by field veterinarians to a veterinary laboratory (Exopol S.L., Zaragoza, Spain) for the etiologic diagnosis of respiratory clinical signs. These samples originated from 210 farms in 32 provinces across Spain and were collected from July 2023 to September 2024 from animals showing respiratory signs such as nasal or ocular discharges, spontaneous coughing, or difficulty breathing. Most of the clinical specimens were pools of up to five samples from the same farm. The sample types included lung or tracheobronchial tissues (*n* = 140 pools), bronchoalveolar lavages (*n* = 104 pools), nasal swabs (*n* = 52 pools) and a mixture of these sample types (*n* = 20 pools). In terms of submission frequency, most farms provided either a single pool (*n* = 111), two pools (*n* = 43), or three pools of specimens (*n* = 31). The remaining farms (*n* = 131) submitted between four to six pools each. For 42 farms, multiple submissions corresponding to independent outbreaks (defined as samples collected more than 3 months apart) were analyzed.

Regarding age distribution, most samples were from pre-weaned calves younger than one month (*n* = 69) and fattening beef calves older than one month (*n* = 175), with fewer samples from replacement animals (*n* = 24) and adults (*n* = 17). The age group was not identified for 31 samples. The sample size from different farms, age groups or time periods could not be controlled, as the samples were submitted for diagnostic purposes based on the criteria of the veterinary surgeons in response to an unusual increase in respiratory disease cases.

### 2.3. Nucleic Acid Extraction and Molecular Identification of Pathogens

Individual and pooled samples were processed for nucleic acid isolation using the MagMAX™ Pathogen RNA/DNA commercial kit (Thermo Fisher Scientific, Waltham, MA, USA). This was performed according to the manufacturer’s instructions on an automated magnetic particle processor (KingFisher Flex; Thermo Fisher Scientific, Waltham, MA, USA). For each nucleic acid sample, the presence of IDV and a panel of other respiratory agents, including BPI3V, BRSV, BVDV, BHV-1, BCV, *Mannheimia haemolytica*, *Pasteurella multocida*, *Histophilus somni*, and *Mycoplasma bovis*, was evaluated using commercial qPCR kits (EXOone qPCR kits, EXOPOL, Pol Río Gállego D/14, San Mateo de Gállego, 50840 Zaragoza, Spain). The manufacturer’s instructions are available at https://www.exopol.com/es/exoone/manuals.php (accessed on 24 September 2025). Briefly, the qPCR and RT-qPCR reactions were conducted on a QuantStudio 5 Real-time PCR machine (Applied Biosystems, Marsiling, Singapore), using a single cycling protocol: reverse transcription at 45 °C for 15 min, enzyme activation at 95 °C for 5 min, and 42 cycles of 95 °C for 15 s and 60 °C for 60 s. Fluorescence was determined in the FAM channel for target detection and in the HEX channel for endogenous control amplification. The data were analyzed using the corresponding software (QuantStudio Design & Analysis software v1.5.2, Thermo Fisher Scientific) and samples with a threshold cycle (Ct) value below 38 were considered positive.

### 2.4. Sequencing of Influenza D Hemagglutinin-Esterase (HEF) Gene

A subset of 13 influenza D virus-positive samples was further investigated to obtain partial sequences of the segment 4 hemagglutinin-esterase (*HEF*) gene. These samples were selected for sequencing based on a Ct value of <32 for IDV detection, and their origin from farms across different provinces (*n* = 11) to ensure broad geographical coverage. Two pairs of primers (IDV-HF-25-F, IDV-HEF1104-R and IDV-HEF-982-F, IDV-HEF-2023-R) previously described were used to amplify two overlapping segments of 1080 bp. and 1042 bp. of the *HEF* gene, respectively [9]. The conventional one-step RT-PCR amplification was performed using the SensiFAST Probe No-ROX One-Step Real-time PCR kit (Bioline Reagents Ltd., London, UK) in a 50 μL reaction mixture. This mixture contained 25 μL of 2 × One Step RT-PCR Buffer, 500 nM of each primer, 1 μL of Ribosafe RNase inhibitor, 0.5 μL of Reverse transcriptase and 15 μL of extracted RNA. The reaction was run on a QuantStudio 5 Real-time PCR machine (Applied Biosystems, Marsiling, Singapore) with the following thermal conditions: a reverse transcription step at 45 °C for 20 min, heating at 95 °C for 5 min followed by a two-step cycle (15 s at 95 °C, 60 s at 58 °C) repeated 40 times. The PCR products were subjected to electrophoresis in 1.5% *w*/*v* agarose gels and visualized with a UV transilluminator. Samples with an expected amplicon size were purified and sequenced in both directions with the same pair of primers used for amplification at STABvida Laboratories (Caparica, Portugal), with an Applied Biosystems 3730 system (Thermo Fisher Scientific, Waltham, MA, USA).

The *HEF* gene sequences were compared to those available in Genbank using Blastn 2.17.0 analysis [30]. Sequences were assigned to their corresponding lineages using the Return Time Distribution methodology and the online IDV tool (https://bioinfo.unipune.ac.in/IDV/home.html (accessed on 24 September 2025)) [31]. The tool requires a minimum length of 1796 nucleotides for reliable assignment (i.e., 90% of the total *HEF* gene length). A Neighbor-joining phylogenetic tree was constructed using Kimura’s two-parameter model in MEGA 11 software (https://www.megasoftware.net; accessed on 24 September 2025) [32]. The robustness of the branching patterns was tested by 1000 bootstrap replicates. Tree drawing was performed online using the iTOL v6 tool [33]. Representative sequences for the five recognized lineages, plus the tentative D/France2012 lineage, were included for branch identification [31]. Nucleotide diversity among IDV strains assigned to different lineages was evaluated using DNAsp software [34].

### 2.5. Statistical Analysis

Seasonality was evaluated by defining four categories: spring (21 March–20 June), summer (21 June–22 September), autumn (23 September–21 December) and winter (22 December–20 March). The strength of co-occurrence between analyzed pathogens was assessed using Cramér’s V test. Associations between the frequency of pathogens (singly or co-occurring with other pathogens) and categorial variables, such as season, age and sample type, were analyzed using the chi-square test. All statistical analyses and graphics were performed using R Studio^®^ (Boston, MA, USA). The level of statistical significance (*p*-value) was set at 0.05.

### 2.6. Nucleotide Sequence Accession Numbers

The sequences of IDV strains from this study were submitted to Genbank with accession numbers PV932148 to PV932162.

## 3. Results

### 3.1. Detection of IDV and Other Pathogens in Cattle with Respiratory Clinical Signs

**Figure 1:**
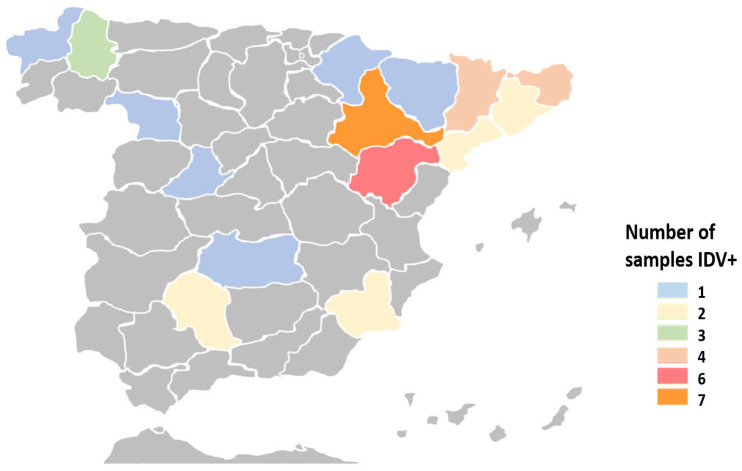
Distribution and number of IDV-positive samples by province in Spain. No samples were submitted from the 35 provinces marked in grey color.

A total of 38 specimens (12%, 95%CI: 8.9–16.1%) tested positive for IDV using the RT-qPCR assay. The mean Ct value for the IDV-positive samples was 29.1, with most of them (*n* = 32) exhibiting a Ct < 35. The detection of IDV was significantly more frequent in bronchoalveolar lavage samples (23/104, 22.1%) and nasal swabs (7/52, 13.5%) compared to lung tissues (7/140, 5%) and a mixture of samples (1/20, 5%) (*p* < 0.05). Positive samples were collected from 30 farms (11.1%) across 15 provinces throughout Spain (Figure 1). Regarding age distribution, most positive samples originated from fattening calves (19/175, 25.3%) and suckling calves (12/69, 17.4%). IDV infection was less common among replacement heifers (1/24, 4.2%) and adults (1/17, 5.9%). However, there were no statistically significant differences in IDV infection prevalence among the different age groups. The age of the animals could not be identified for five positive samples. Although IDV-positive specimens were detected throughout the year, their frequency was significantly higher in winter (12/59; 20.3%) and spring (12/61; 19.7%) compared to summer (7/83; 8.4%) and autumn (7/113; 6.2%) (*p* < 0.05).

Out of the 316 samples collected from cattle with respiratory signs, 252 (79.7%) tested positive for at least one of the pathogens analyzed. The prevalence of the other viral and bacterial pathogens is shown in Table 1. The most prevalent viruses identified in this study were BCoV, BRSV and BPI3V, while among the bacteria, *M. bovis* and *P. multocida* showed the highest frequencies, being detected in more than half of the tested samples. All IDV-positive samples exhibited co-infections with other pathogens. Specifically, 36 of the 38 (94.7%) IDV-positive samples harbored a total of 3 to 7 viral and/or bacterial pathogens. The most prevalent co-infections with IDV were found with *M. bovis* (*n* = 35), *P. multocida* (*n* = 32), *M. haemolytica* (*n* = 24), *H. somni* (*n* = 12), BCov (*n* = 21), BPI3V (*n* = 13), BRSV (*n* = 10), Pestivirus (*n* = 7) and BHV-1 (*n* = 1).

**Figure 2:**
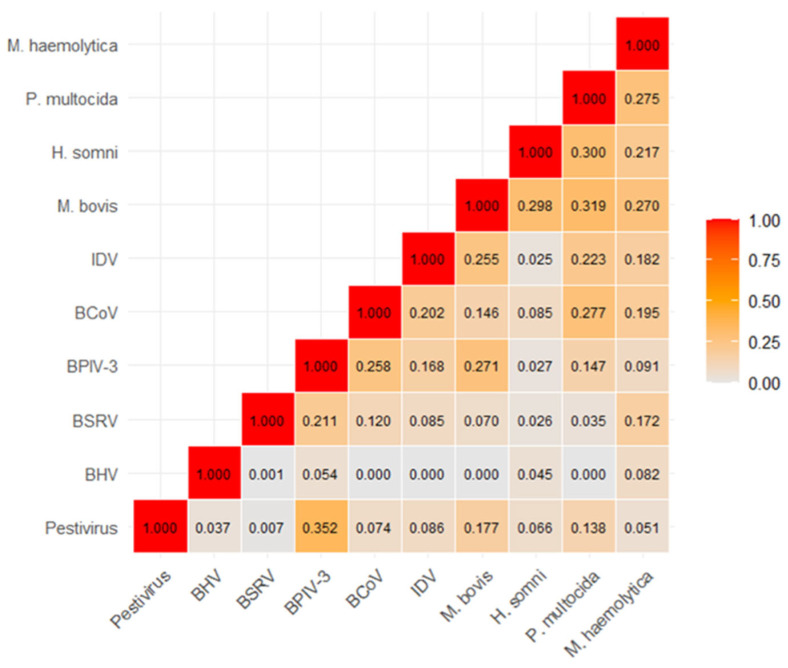
Cramer’s V coefficients measuring the strength of association between influenza D virus (IDV) and the other analyzed respiratory pathogens in this study. Higher coefficient values and a darker color code indicate a stronger co-occurrence between the pathogens.

Cramér’s V analysis revealed a highly variable strength of association among the rest of the pathogens (Figure 2). The strongest association was observed between Pestivirus and BPIV-3 (V = 0.352). Conversely, several viral associations were found to be negligible (V < 0.05), most notably between BHV-1 and BSRV (V = 0.001). Moderate associations (V > 0.250) were also notable within the bacterial group, including *M. bovis* with *P. multocida* (V = 0.319) and *H. somni* (V = 0.298). IDV infection exhibited its strongest co-occurrence with *M. bovis* (V = 0.255), followed by moderate ties with other major bacterial pathogens, notably *P. multocida* (V = 0.223). Conversely, IDV co-occurrence with key viral agents like BHV-1 (V = 0.000) was negligible.

### 3.2. Sequencing and Phylogenetic Analysis

**Figure 3:**
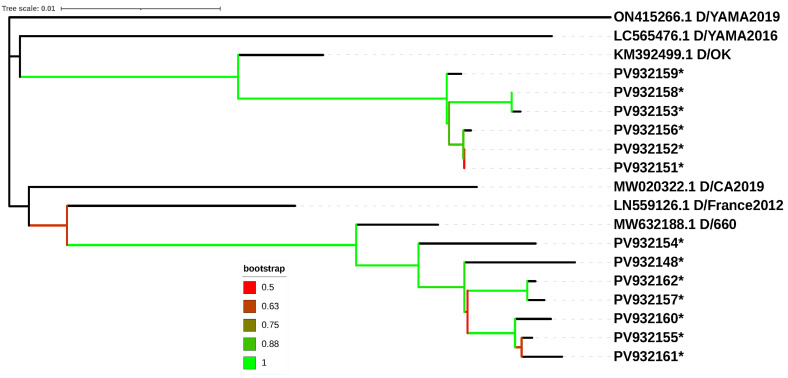
Phylogenetic relationships of IDV lineages determined by Neighbor-Joining analysis of partial *HEF* sequences (1003 nucleotides). This tree includes representative sequences for the five established IDV lineages and the tentative D/France2012 lineage. Sequences generated in the present study are highlighted with an asterisk (*). All entries include their corresponding GenBank accession numbers.

A total of 13 *HEF* gene sequences were assigned to two different lineages using the online IDV tool [33]. Six sequences (PV932151, PV932152, PV932153, PV932156, PV932158 and PV932159) belonged to the D/OK lineage, while five (PV932148, PV932154, PV932155, PV932157 and PV932160) belonged to the D/660 lineage. Two sequences (PV932161 and PV932162) could not be reliably assigned by the IDV tool due to their relatively short length; however, they clustered with the D/660 lineage in the Neighbor Joining analysis. The Neighbor-Joining tree (Figure 3) was consistent with the IDV tool’s assignments where comparisons were feasible.

Genetic variability among the Spanish isolates of the D/OK lineage was relatively low, as calculated using DNAsp [34]. Out of 1877 sites, only 13 (0.7%) were variable. Notably, two sequences (PV932151 and PV932152) were identical, resulting in five distinct haplotypes among the six D/OK sequences. The haplotype diversity index was 0.933, and the nucleotide diversity index (Pi) was low (0.00334). The mean number of nucleotide differences among the six sequences in this D/OK lineage was 6.267.

In contrast, the D/660 lineage exhibited higher genetic diversity. Among the 1876 nucleotides available, 58 (3.1%) were variable, which is approximately four times higher than in the D/OK lineage. The D/660 sequences generally showed greater diversity, as indicated by a haplotype diversity index of 1 (all five sequences were unique), a nucleotide diversity (Pi) of 0.01 (approximately three times higher than D/OK) and a mean number of nucleotide differences of 19.095 (also about three times higher than D/OK).

Blastn analysis of the obtained sequences revealed high degree of homology not only with bovine isolates but also with swine sequences. Specifically, a strong genetic link was detected between our bovine IDV isolates and sequences from swine. The D/OK *HEF* sequences showed maximum similarity (100% coverage, >99% similarity) with a swine isolate from Luxembourg (GenBank: ON038934.1). Similarly, the D/660 isolates exhibited high similarity (>99%) with a French swine isolate (PP133485.1).

## 4. Discussion

Influenza D virus has been implicated in respiratory disease in young cattle, often exacerbating coinfections within the BRD complex and increasing overall disease severity [10,11]. Furthermore, due to its recognized zoonotic potential, IDV poses a potential occupational health risk for cattle workers as an emerging pathogen [35]. In the present study, IDV infection was detected in a significant proportion of animals from Spanish cattle farms distributed across various regions of the country experiencing BRD outbreaks. Specifically, 12% of samples originating from 11.1% of farms tested positive using RT-qPCR. IDV was identified in both the upper (nasal swabs) and lower (bronchoalveolar lavage) respiratory tracts. Infection was notably more common among young animals, particularly in suckling and fattening calves, with one in four of the latter testing positive. These results suggest that IDV should be included in routine diagnostic respiratory panels. A limitation of this study is the inherent selection bias of the retrospective diagnostic sample set. Also, the use of sample pooling could have resulted in some false-negative results, so the IDV incidence calculated in the present work could be underestimated. Nevertheless, the findings still provide the first evidence of IDV circulation and preliminary characterization of this emerging agent in Spanish cattle.

Several serological and molecular studies have confirmed the presence of IDV in cattle across Europe, suggesting its enzootic status and consolidating the role of cattle as the primary host of the virus [24,25,26,27,36]. Coinfections with other respiratory pathogens are very frequent in IDV-infected cattle, often involving more than two or even three distinct agents, which significantly complicates control and treatment strategies [10,37].

Our findings establish that IDV was more prevalent in the sampled population than several well-recognized viral pathogens, including Pestivirus (BVDV) and Bovine Herpesvirus 1 (BHV-1), both of which are known for their substantial pathogenicity in cattle [38]. This higher prevalence underscores the growing recognition of IDV as a significant viral component of the Bovine Respiratory Disease (BRD) complex. Importantly, the majority of IDV-positive samples exhibited multiple-pathogen presence, harboring at least two additional pathogens. The bacteria *M. bovis* and *P. multocida* were notably prevalent, being detected in nearly all IDV-positive specimens. Furthermore, Cramér’s V analysis provided crucial insight into the strength of these co-infections. IDV exhibited its strongest association with *M. bovis* (V = 0.255), followed by *P. multocida* (V = 0.223). This positive association suggests a potential synergistic relationship, where the initial viral event by IDV may predispose the host to colonization and proliferation by these key bacteria. Co-infections with other respiratory viruses were also common, with BCoV and BPIV-3 detected in 55.3% and 34.2% of IDV-positive specimens, respectively. Conversely, IDV co-occurrence with certain pathogens, such as *H. somni* (V = 0.025) and BHV-1 (V = 0.000), was found to be negligible. In the case of BHV-1, the low frequency of its detection in the overall study population must be considered, as this limits the statistical power to establish a significant association. These results corroborate recent findings that highlight the high frequency and complex nature of co-infections in BRD outbreaks, emphasizing the need for continued investigation into the implications of such intricate viral–bacterial interactions [29,39].

While Bovine Respiratory Disease (BRD) can occur throughout the year, particularly in young calves and feedlots, winter is generally considered a peak season for respiratory viruses, notably for Bovine Respiratory Syncytial Virus (BRSV) [2]. Previous cluster analysis of BRD-associated pathogens in Spain (2020-2021) reported seasonality in outbreaks linked to four viruses (BPI3V, BRSV, BCV and BVDV), which were more frequently detected in cold months (December to March). In contrast, no seasonality was observed in a second cluster characterized by less prominent viral involvement; notably, IDV infection was not analyzed in that study [39]. IDV-positive specimens were detected year-round in the current study, even if the frequency was significantly higher during the winter and spring, suggesting that infection peaks during the colder and transition periods. However, a more extended study period is warranted to fully assess the possibility of a cyclic pattern.

The two major genetic lineages of IDV detected so far in cattle in Europe, D/OK and D/660, were found in a similar proportion of samples in the current study. Prior surveillance indicated that D/OK was the most frequent lineage circulating in cattle in Europe until the earliest D/660 strain was detected in Italy in 2018 [25,26]. Subsequently, a lineage shift from D/OK to D/660 has been increasingly reported, along with the detection of re-assorting strains containing gene segments from both lineages in countries including Italy, Denmark, Sweden and France [23,26,28,29,40]. Since our analysis focused solely on the *HEF* segment sequence, we could not verify the existence of specific lineage shifts leading to re-assorted isolates in our samples.

The lower genetic variability observed among D/OK isolates compared to D/660 in the current study suggest a shorter divergence time for the D/OK lineage within our sample set. Despite the considerable geographic distance between both countries, BLASTn alignment showed high similarity (98.90% to 98.92%) between the present Spanish D/OK *HEF* sequences and those reported in Sweden [29], further suggesting a relatively short divergence time. The slightly lower similarity observed when comparing our data to the older Danish data is likely attributable to the temporal difference in sample collection (2018–2020 vs. 2024) [28]. Our analysis revealed a strong genetic link between our bovine IDV isolates and sequences from swine. This finding strongly suggests potential inter-species transmission (spillover) between swine and cattle, a phenomenon previously confirmed experimentally [41]. Although IDV is primarily considered a bovine pathogen, the high similarity with swine sequences found across Europe emphasizes the necessity for strict epidemiological surveillance of live animal movements to control inter-species dissemination.

## 5. Conclusions

Results of this study demonstrate the emergence and active circulation of influenza D virus (IDV) in cattle farms throughout Spain. Our findings suggest IDV is more prevalent than other well-recognized respiratory viruses, such as BVDV or BHV-1. Furthermore, the high rate of co-infection—with all IDV-positive specimens harboring four to seven viral and/or bacterial pathogens—underscores the complexity of respiratory disease on these farms. The co-circulation of the D/OK and D/660 lineages was confirmed. The high genetic homogeneity observed among our D/OK *HEF* sequences suggests a recent divergence and rapid continental dissemination of this lineage across Europe. This study represents the first report of IDV infection in cattle in Spain.

## Figures and Tables

**Table 1:** Prevalence of analyzed pathogens in cattle showing respiratory clinical signs in Spain (*n* = 316).

	Positive Samples (%)		
	Pathogen as Sole Agent	Infections with One or More Pathogens	Total
IDV	0	38 (12%)	38 (12)
BCoV	3 (0.9)	92 (29.1)	95 (30.1)
BHV-1	1 (0.3)	9 (2.8)	10 (0.3)
BRSV	4 (1.3)	52 (16.5)	56 (17.7)
Pestivirus	1 (0.3)	32 (10.1)	33 (10.4)
BPI3V	1 (0.3)	51 (16.1)	52 (16.4)
*H. somni*	0	86 (27.2)	86 (27.2)
*M. bovis*	15 (4.7)	179 (56.6)	194 (61.4)
*M. haemolytica*	5 (1.6)	120 (38)	125 (39.5)
*P. multocida*	9 (2.8)	167 (52.8)	176 (55.7)

## Data Availability

The data presented in this study are available in Genbank. [https://www.ncbi.nlm.nih.gov/genbank/, accessions PV932148 to PV932162]. (accessed on 24 September 2025).
